# ‘Just because she’s young, it doesn’t mean she has to die’: exploring the contributing factors to high maternal mortality in adolescents in Eastern Freetown; a qualitative study

**DOI:** 10.1186/s12978-018-0475-x

**Published:** 2018-02-21

**Authors:** Lucy November, Jane Sandall

**Affiliations:** Division of Women and Children’s Health, Faculty of Life Sciences and Medicine, Kings College London, St Thomas’ Campus, Westminster Bridge Road, London, SE1 7EH UK

**Keywords:** Sierra Leone, Adolescent, Pregnancy, Transactional sex, Abandonment, Delayed care-seeking, Maternal death, Mentoring, Blood donation

## Abstract

**Background:**

In Sierra Leone, 34% of pregnancies and 40% of maternal deaths are in the adolescent population. Risks are known to be higher for younger adolescents, this being borne out by a household survey in Eastern Freetown in 2015. This current qualitative study, funded by Wellbeing of Women’s international midwifery fellowship, was conducted to explore the causes of this high incidence of maternal death for younger teenagers, and to identify possible interventions to improve outcomes.

**Methods:**

This qualitative study used semi-structured interviews (*n* = 19) and focus groups (*n* = 6), with a wide range of professional and lay participants, recorded with consent. Recordings were transcribed by the first author and a Krio-speaking colleague where necessary, and Nvivo software was used to assist with theming of the data around the three main research questions.

**Results:**

Themes from discussions on vulnerability to teenage pregnancy focused on transactional sex, especially for girls living outside of their birth family. They included sex for school fees, sex with teachers for grades, sex for food and clothes, and sex to lessen the impact of the time-consuming duties of water collection and petty trading. In addition, the criminal justice system and the availability and accessibility of contraception and abortion were included within this major theme. Within the major theme of vulnerability to death once pregnant, abandonment, delayed care seeking, and being cared for by a non-parental adult were identified. Several obstetric risks were discussed by midwives, but were explicitly related to the socio-economic factors already mentioned. A cross-cutting theme throughout the data was of gendered social norms for sexual behaviour, for both boys and girls, being reinforced by significant adults such as parents and teachers.

**Conclusion:**

Findings challenge the notion that adolescent girls have the necessary agency to make straightforward choices about their sexual behaviour and contraceptive use. For girls who do become pregnant, risks are believed to be related more to stigma and abandonment than to physical maturity, leading to lack of family-based support and delayed care-seeking for antenatal and delivery care. Two potential interventions identified within the research are a mentoring scheme for the most vulnerable pregnant girls and a locally managed blood donation register. A feasibility study of a pilot mentoring scheme is currently underway, run by the first author and a local partner.

## Plain English summary

In Eastern Freetown, Sierra Leone, young pregnant teenagers have a much higher risk of death than older women. This study was carried out to find out why, and what interventions might improve outcomes for this vulnerable group. The first author conducted focus groups and interviews with a wide range of professionals and lay participants, and analysed the responses and ideas generated into themes, based on the three main research questions.

Themes from discussions on ‘vulnerability to teenage pregnancy’ focussed on transactional sex; for school fees, sex with teachers for grades, and sex for food and clothes, as well as issues about the criminal justice system and the availability and accessibility of contraception and abortion. Under the heading ‘vulnerability to death once pregnant’, abandonment, delayed care-seeking, and being cared for by a non-parental adult were identified. Several risks relating to childbirth were discussed by midwives, but they were clear that these were because of the socio-economic factors already mentioned. A theme picked up throughout was that both girls and boys have behaviours modelled to them by adults such as parents and teachers which perpetuate particular ways of behaving, especially regarding sexual behaviours.

Findings challenge the notion that teenage girls have real choices regarding their sexual behaviour and contraceptive use, and suggest that risks to pregnant girls are because of stigma and abandonment, rather than physical immaturity. Two potential interventions are a mentoring scheme for the most vulnerable pregnant girls and a locally managed blood donation register. A pilot of the mentoring scheme is currently underway.

## Background

Sierra Leone has an estimated maternal mortality ratio (MMR) of 1165 maternal deaths per 100,000 live births, the highest in the world [1]. It is also one of only six countries in Sub-Saharan Africa (SSA) where more than 10% of girls become mothers before the age of 16, with increased MMRs for adolescents in general, and particularly high ratios for the under-16 age group when data is disaggregated by age [2]. Overall, 34% of all pregnancies and 40% of all maternal deaths occur amongst teenage girls [1]. In response to this, in 2013, the Government of Sierra Leone (GoSL) drew together a multi-agency and cross-ministry collaboration and developed the National Strategy for the Reduction of Teenage pregnancy (hereafter referred to as ‘the national strategy’) with six key pillars of action [3].^1^

Sierra Leone experienced 10 years of brutal civil war which finally ended in 2001, by which time the health system was barely functional and under-used due to charges at point of care. However, in 2010, a new policy of free health care for pregnant and lactating women and children under five was introduced [4], and use of health services increased significantly; in the 5 years from 2008 to 2013, the proportion of women giving birth with a skilled birth attendant rose from 42% to 60% [1, 5]. Sadly, the Ebola Virus Disease (EVD) outbreak in 2014/15 was another devastating onslaught for the fragile health system, with 7% of health workers dying from the disease compared with 0.06% of the general population [6].

Progress in addressing the issue of teenage conception was stalled by the EVD outbreak, and during this time a combination of closed schools and increased poverty is thought to have led to a significant increase in the numbers of teenage pregnancies [7]. In the aftermath of the epidemic, the national strategy was revised and updated and was due to be relaunched in 2017, though to date has not been.

The current study came about following a local household survey conducted in 2015 in Kuntorloh, a suburb of the capital city, Freetown, by a community-based organisation (CBO), Lifeline Nehemiah Projects (LNP), due to a perception in the local community that the local MMR was significantly higher than the 1165 per 100,000 presented in the 2013 Sierra Leone demographic health survey (SLDHS). The first author had lived in Freetown and worked with LNP from 2001 to 2004 as a community health educator, and in 2015 partnered with the LNP team in a consultancy role to carry out this local survey with a team of volunteers. Data were collected on 1400 pregnancies and analysed by the first author. The overall MMR was indeed elevated, at three times the national figure. However, the data for teenagers was even more concerning, with 1 in 7 of the pregnant teenagers under 17 dying from a maternal cause (the first author’s unpublished data). Despite sample-size limitations, this merited further exploration. The first author was funded by Wellbeing of Women’s international midwifery fellowship to carry out this qualitative study with the aim of better understanding the factors which put younger women at greater risk of maternal death, in order to work with local people to develop and evaluate interventions to reduce these risks.

## Methods

### Study design

This is a qualitative study using focus groups and semi-structured interviews carried out during two field trips to Freetown in 2016/17. Groupings for focus group discussions were selected to gain the maximum insight from participants’ varied perspectives, whilst working within the field trips’ limited time frames. The six focus groups comprised: two groups of ten adolescent mothers, a group of nine young men (aged 19 to 29), ten midwives working in the main referral hospital, eleven health workers including midwives from two peripheral health units (PHUs), and a discussion with two secondary school teachers. Semi-structured interviews were conducted with three senior midwives (research, education and clinical), a staff-grade midwife from a PHU, two consultant advisors from UNICEF and UNFPA, three GoSL senior advisors (two from the Ministry of Health and one from the Ministry of Social Welfare), a country director and project lead for two different international non-governmental organisations (NGOs), a director of a CBO, three local women’s leaders, a senior community leader, and a teenage mother who could not attend the focus groups but was keen to talk about her experience.

### Recruitment and procedures

The primary inclusion criterion for the study was personal, family, or professional experience of teenage pregnancy, with purposive sampling as the first strategy. One group of young mothers was recruited within a local vocational skills training institution (not LNP); the second group of ten young mothers was recruited by invitation from a community worker; and the nine young men were recruited from the volunteers who had previously taken part in the household survey in 2015 and were known to the first author. No young mothers who were invited declined to be involved; it could be argued that this indicated coercion, however every effort was made to assure participants that participation was voluntary. This is discussed more fully within the ethical considerations.

Midwives were invited by two key informants; one within the referral hospital and one working in a local PHU. Other community participants were recruited via snowball sampling, where an existing participant suggested a new participant based on their knowledge of the circumstances or interests of the prospective participant. In the same way, some professional participants with a clear remit within the teenage pregnancy arena were contacted prior to the research visits via email request, and others were contacted and recruited by email or telephone during the research visit, having been suggested by other participating professionals. Teachers, CBO and NGO participants were recruited through personal contacts. All interviews and focus groups were conducted by the first author and her Sierra Leonean research assistant, a female LNP staff member with a master’s qualification in gender studies, in either English or Krio, the commonly spoken local language with which the first author is very familiar but not fluent. Focus groups were conducted in various community settings at times when discussions could not be overheard, and no other non-participant observers were present. Interviews were conducted at settings chosen by the participant, and some interviews included the participant’s junior colleagues. Most of the focus groups and interviews were between 40 and 70 min duration.

### Ethical considerations

Participants were sent or given participant information sheets in the days prior to the interview or focus group where possible. Where this was not possible, the information sheet was given or read to the participant in English or in Krio prior to the interview or focus group, with opportunity to ask questions prior to giving or withholding consent. It was stressed that participating in the study was voluntary, and would not advantage or disadvantage the participant in any way. This was particularly important for the adolescent mothers and young men in the study, as they were also potential beneficiaries of various community-based provision such as LNP’s vocational training, and knew of the first author’s association with LNP. Where applicable, it was clearly explained that their participation was confidential and would not be disclosed to LNP staff. The information sheets included issues of anonymity, data security and that they could withdraw themselves and their data from the study within specified time frames.

### Research questions

This qualitative study set out to answer three key questions about the high rate of maternal mortality amongst adolescent girls in Freetown:

- What are the factors which increase vulnerability to teenage pregnancy?
- What are the vulnerabilities which increase the risk of death from pregnancy-related causes in adolescents?
- What could be done to mitigate the risks associated with teenage pregnancy in this part of Freetown?

### Data analysis

Most interviews and focus groups were recorded. Three of the government or UN agency interview participants declined to be recorded; notes were taken during these interviews which were used as personal observations. Recorded data was listened to and transcribed primarily by the first author and a Krio-speaking transcriber where applicable, who also clarified any Krio words or phrases not familiar to the first author. The data was then transferred to Nvivo which was used to assist with coding of the data into major themes categorised around the research questions. Though this study was underpinned by grounded theory, using an inductive approach to explore themes being identified from the data in an iterative manner, the time and opportunity constraints of limited field trips meant that not all the themes were entirely saturated. For example, when the subtheme ‘sex for grades’ was identified from a focus group with teachers, a true grounded theory approach would have led to participants in policy roles being re-interviewed to seek their views on issues such as prosecutions of teachers; unfortunately, this was not possible. In contrast, for other subthemes such as issues around blood donation, because it was identified on the first day of data collection, it was possible to hear from all subsequent participants and therefore reach the point where no new information or opinions were being expressed. Because this study used a strong partnership approach with LNP, and feedback was sought on the author’s interpretation of the data, the validity of the findings is strengthened.

## Results

The data from this study falls under the three major themes in line with the three key questions.

Sub-themes emerged under each major theme, and the following table summarises these. For each subtheme, the main data sources are outlined. Participant codes are given for quotations. It was not possible during transcription to identify individuals within focus groups, hence group coding rather than individual coding is given. The exception was the teachers’ focus group which only had two participants (T1 and T2). FG denotes source as a focus group; I denotes source as an interview.

**Table:** 

Major theme related to research question	Subthemes within major theme	Key informants on this subtheme
Vulnerability to teenage pregnancy	Transactional sex • ‘not their own child’ • ‘water for water’ • ‘sex for school fees/grades’ • the criminal justice system Contraception and abortion	• young men’s focus group (YMFG) • women’s leaders (WL) • teachers’ focus group (T1 and T2) • Government of Sierra Leone participants (GI) • Senior NGO workers (NGO)
Vulnerability to maternal death once pregnant	Abuse and abandonment • ‘just because she’s young it doesn’t mean she has to die’ Increased birth complications • Delayed care seeking Risk of death from anaemia and PPH	• young mothers’ focus groups (MFG) • hospital midwives’ focus group (HMFG) • community clinic health workers focus group (HWFG) • senior midwives (SM) • senior community leader (SCL)
Possible interventions to reduce maternal death in adolescents	Mentoring Community-based blood donation • compensation • blood donation experience	• women’s leaders • young men’s focus group • midwives and other health workers

### Vulnerability to teenage pregnancy

By definition, teenagers who do not become pregnant cannot die from maternal causes. For this reason, it is valid to include within the results, analysis and discussion, factors which lead to teenage pregnancy alongside factors which lead to maternal death in pregnant teenagers.

#### Transactional sex

Despite use of language such as ‘being in love’, the context for much of the sexual activity discussed in this study appears to define it as transactional; as a way of minimising the burden of the time-consuming duties of petty trading or water collection; for money to pay for school fees and other expenses; and as a condition proposed by school teachers for girls to pass exams or be promoted to the next school year. The lack of adult care and financial provision for girls who were living with extended family instead of their birth family seemed to exacerbate their exposure to all of these risks, although girls living within their own birth families were also under pressure to have transactional sex.

##### ‘Not their own child’

Many households in Freetown include children and young people who are family relatives or unrelated to the family, as well as children born into the household. Often these additional children are sent from rural Sierra Leone to attend school or as a way of redistributing the economic burden when part of a family falls into poverty, due for example to the death of one parent.

When asked what makes some girls more vulnerable to pregnancy and maternal death, many participants proposed that living in a household other than the one into which you were born is a key vulnerability, citing ‘lack of proper care’ and ‘no-one to guide them’. Many girls in this situation are sent out to sell goods, either for their household, or to earn money to support themselves and pay for schooling if this need is not being met for them, risking exploitation:

*The other thing is the trade the girls do from house to house. When they are hawking their trade, some older men will call to them saying ‘Come! I want to see or buy what you are selling’. He will call her into the house and say, ‘I will buy everything you are selling if you have sex with me.’ (WLI3)*

And girls are faced with complex decisions with lose-lose outcomes:

*Some go to school in the afternoon so in the morning they will have to do their trade before school. At a certain time before noon they will come home and get ready for school … (it can) take the whole day to sell and when a man offers to buy everything she has for sex, that can be tempting. (WLI2)*

##### ‘Water for water’

There are two seasons in Sierra Leone; the rainy season from around April to October, and the dry season from around November to March. Poor communities such as Kuntorloh have no water delivery infrastructure; in the rainy season water is harvested from roofs, but in the dry season water must be collected from community wells, which gradually dry up as the season progresses. Water collection is one of the time-consuming domestic duties which young people, particularly teenage girls, are responsible for; a task which can take five or six hours of queuing at the height of the dry season. Both research visits took place within the dry season, and participants were keen to talk about the risks associated with water collection, explaining that the queue can be bypassed by girls having sex with the youths who run the wells. A local expression ‘water for water’ was cited by several of the participants:

*Teenagers get pregnant a whole lot because of the water crisis – ‘if you give me water, I’ll give you sex’. ‘Water for water’. Especially in February, March, those two months. Oh! Very tedious – water! (WLI1)*

Participants clearly viewed this sex as transactional rather than coerced or forced and it was clear that this phenomenon was not an example of the risks cited in the literature about being raped whilst walking to the water points [8].

*It’s an agreement, as they are in love. It’s not a rape. The girls do not want to waste too much time at the stream or well. So, if the boy who controls the well or the water tap is the girl’s boyfriend... even if she has twenty or even fifty containers, he will make sure her containers are filled up first. So the girls get home a little bit earlier than normal. (WLI2)*

For girls who are trying to attend school and study for exams this is a real dilemma:

*Interviewer: what if a girl decides that she is not going to have sex?*

*(WLI3): you will stand there for the rest of the day you would not be able to get the water.*

This participant, an older woman, expresses her frustration at the infrastructure deterioration which has allowed this problem to develop:

*The dam has got to be repaired or worked on. In days gone by here in Freetown there used to be taps at every street junction. Then we never used to have this problem of young girls getting pregnant due to water crises. (WLI3)*

Clearly, the task of collecting water during the dry season can present girls with difficult choices in terms of sexual behaviour, particularly in terms of freeing up time for study and school attendance.

##### Sex to pay for school fees and hidden charges, and school-based financial abuse

Sierra Leone does not provide free secondary school education; all families expect to pay school fees and many plan accordingly. However, it is the unpredictable, hidden costs which put additional strain on teenagers. Some of these charges are for ‘extra classes’, where teachers only partially cover the syllabus during regular school hours so that students are forced to pay directly to teachers to supplement normal school. Young people who have to source this money for themselves employ a range of strategies to do so, including girls engaging in transactional sex with older men, or ‘sugar daddies’:

*The man that impregnated me was helping me a lot for my schooling, so I fell in love with him, but I was so small at the age of fourteen. (MFG).*

Other more blatant financial abuse occurs when teachers require additional payments in exchange for a student passing an exam or being promoted to the next school year. This study describes a deeply entrenched system of extortion for grades and progression levied by individual teachers, both male and female:

*It’s just like bidding. The highest bidder has the highest grade. If you give 10 000 Leones, you will have your 10 000 Leones grade. If somebody comes with 20 000 Leones, that person will automatically have a higher grade than you! Let’s say for example Salimatu comes to school, and she does not have that money. Automatically she will have to repeat (the school year). No matter how brilliant Salimatu is, she will have to repeat that class simply because she does not have that money to satisfy the teacher. You have to satisfy your teacher both in writing and in the financial aspect. (T1)*

School attendance presents teenagers with a raft of financial challenges, even if their basic school fees are being paid. Having to find the extra money needed to access all of the curriculum, to ensure that assignments are graded or even to pay what are essentially bribes to teachers to pass exams and progress through school can put girls under pressure to have transactional sex with older men who can provide for them*.*

#### Sex for grades

An even more direct risk associated with schooling is the sexual abuse perpetrated by teachers whereby they arrange to have sex with female students in exchange for academic achievement [8–11]. This is identified as a pervasive issue, but one which young people are reluctant to discuss [8].

In the current study, this was borne out by the issue being discussed exclusively by professional participants rather than by the adolescents themselves. The practice appears to undermine the whole system upon which academic success is based:

*Children who are not able to read or write and find it difficult to, you know, understand questions and pass exams … some male teachers exploit that situation by offering, asking them to offer sex for grades. (T2)*

This seems to be a ubiquitous issue. The following refers to one of the most highly regarded girls’ schools in the country:

*The other day my daughter was telling me ‘Mama, even when I’m submitting my assignment, my teacher is asking me for money.’ If you don’t give your child enough money to pay for the assignment, what happens? ‘Ah Mr X, I don’t get money’. ‘OK, meet me at my house’ ... If the girl doesn’t get pregnant, its infection. STI, HIV. That’s why HIV is as high as it is*. (NGO1)

Whilst some of the sexual activity between older girls and teachers is initiated by the girls themselves, the data also demonstrates a deliberate targeting of very young girls, with evidence of abuse of girls in very early puberty:

*Even in primary school, you see these girls have grown breasts, and maybe you are thinking ‘I don’t want to go to bed with this girl’, but your hands … you may want to like... touch her breasts, her buttocks, in that sexual way, after the class. (T1)*

The evidence points towards this abuse being entrenched within the education system, with the implication that some teachers go into the profession to have easy sexual access to young girls:

*One time when I was at the teacher training college, they asked us to give our names and school of choice for our teaching practice. And some of our colleagues gave their names for a female school for one reason ... the reason is to have more girls you see. There are schools who are marked as ‘sex schools’. Sex schools! The other day someone was telling me ‘ah that school over there. If you go there, you can have women until your tire.’ (T1)*

The issue appears to go beyond individual teachers with reports of ‘sex for grades’ being accepted and even encouraged by school leaders. Participants also noted that, at times, boys are paid to investigate girls’ backgrounds as potential targets for coerced sex by teachers:

*Some of these teachers, they use some of these boys to investigate these girls. Like for example they will say ‘Souri, I really want that girl. Go and look at the background of that girl. If they have any person that is strong in the family.’ And Souri will now go to the girl, and interview the girl. If he says they are poor, then automatically the teacher will take that as an advantage. (T1)*

Various methods are used by teachers to pursue and control girls such as buying the girl a mobile phone, which she is then expected to use to send sexually explicit photos. This study data would indicate that this practice is commonplace, but that the taboo around it is stronger than the ‘sex for school fees’ issue.

It seems apparent that for teenage girls in Sierra Leone, graduating from secondary school is not a simple matter of completing assignments on time and studying for exams. On the contrary, for some young women, pursuing an education seems to be a minefield of risk and difficult decisions where the benefits of an education have to be weighed against the risks of pregnancy, infection and the trauma of unwanted sex.

#### The criminal justice system

With pressure from groups such as Legal Access through Women Yearning for Equality Rights and Social Justice (LAWYERS), legislation is in place in Sierra Leone to address underage sex and child abuse, and it is the first pillar of the 2013 teenage pregnancy reduction strategy. Despite this, prosecutions, whether of family members or professionals like teachers, are rare. In the literature [8, 10], and throughout this research, two common scenarios emerge; of families negotiating a financial settlement themselves, and of families reporting the issue to the police but this process being undermined by senior community members pleading that the man be released for his ‘good character’.

*I think on one or two occasions, I’ve heard of the teacher being taken to the police. But … all the senior members of the society will come; ‘Oh, let this man go, he is a teacher. He’s actually doing very well in this community. Please don’t let this teacher go down to prison.’ (CBOI)*

This senior community leader expressed frustration with the deficiencies in the legal system, proposing a zero-tolerance approach:

*Even the government are unable to … interpret and implement the law properly. When someone has done something bad they should be in prison. If someone dies in pregnancy the person responsible should be charged with murder. If this is done to one, two or three persons as a sample, the others will be afraid. But most of those who commit such offense are freed one after the other. (SCLI)*

However, the picture is not entirely bleak. The national strategy is clear in laying out the legislative framework around child rights and gender-based violence, providing a common understanding of the way ahead, and some NGOs are building on this platform, providing training for community leaders on identifying and dealing with child abuse. The director of one CBO explained how he feels things are changing:

*In the past, there was lots of compromising on sexual abuse cases … but in the past three months, we have supported the prosecution of up to four or five sexual abuse cases of children between the ages of eleven and thirteen. All of them, when these cases are in court, the perpetrators have been remanded in prison. (CBOI)*

There was also evidence that attitudes around abuse by professionals may be starting to change; examples were cited of recent imprisonments of policemen who had been prosecuted for child abuse. However, even though the will to implement legislation may be changing, capacity is a very real barrier; NGO and GoSL participants highlighted the lack of resources as an additional barrier in rural areas:

*In certain far remote communities, where you do not have magistrates court sittings … if a victim is staying in the community which is about 50 or 100 miles to where the court is, without all of this support, transportation, shelter, then she definitely will not come. (GI1)*

Despite a legislative framework being in place to deal with sexual abuse, there currently appear to be a number of barriers to fully implementing this legislation, including a lack of political will to take the issue seriously, a lack of logistical infrastructure to facilitate trials and prosecutions, and a lack of support to girls who choose to put their heads above the parapet and challenge the inherent gender norms and power imbalances to which society generally and the education system specifically are subject.

#### Contraception and abortion

Access to contraception is recognised as a vital component in reducing adolescent pregnancy and maternal death [12–14], and an important part of the national strategy is to make contraceptives more accessible, available and affordable for adolescents. There is work in progress to train health workers in family planning methods, including implants, and to make government clinics adolescent-friendly by allocating trained staff to treat teenagers, with, in some clinics, a separate room to accommodate them. Marie-Stopes and other NGOs are also popular providers. Health workers made a distinction between Freetown and the provinces in regard to contraceptive services, saying that they are well stocked with contraceptives in Freetown, but this is not always the case in the more remote rural areas. Despite availability, health workers identified ongoing stigma as the major reason why girls do not access family planning in Freetown.

Though implants, known in Freetown as ‘captain bands’ have increased in popularity, with younger girls there is a concern about being ‘found out’ due to the visibility of the implant, particularly in the few days after insertion. There was also evidence of myths and taboos around contraceptive use:

*They listen to the people in the street that says it gives cancer, so they’re afraid. (HWFG)*

However, as the midwives pointed out, child spacing for adolescents is particularly important to allow them to finish developing. A major concern amongst the midwives was that girls who were too ashamed to use the clinics often turned to unqualified suppliers whom they referred to as ‘quacks’. This often also included performing unsafe abortions, which the midwives perceived to be a significant contributor to maternal mortality, although rarely counted as such:

*And not only are many of them dying of childbirth, they are dying of abortion. Most of the mothers, because of the embarrassment and everything, take them for abortion. And they die, and they don’t talk about it. (MWI)*

Several of the young mothers described methods they had used to attempt an abortion:

*In the village, when I knew that I was pregnant, I drank a lot of herbs to destroy the pregnancy. (MFG1)*

*I drank loads of Seven-up with blue clothes dye in it. (MFG1)*

And several told of how boyfriends or parents had tried to persuade them to have an abortion, but they had refused or avoided it.

Despite significant advances in the supply and range of contraception available to women in Sierra Leone, there are persistent issues of availability in rural areas, and accessibility for stigmatised groups such as adolescents. The use of unregulated contraception and abortion put these younger women at additional risk.

### Vulnerability to maternal death once pregnant

Many women in Sierra Leone have had a child in adolescence. As mentioned, the country has a very high teenage pregnancy rate, and one might reasonably conclude that this would normalise and reduce the stigma associated with teenage pregnancy. This study indicates that this is not the case, with teenage pregnancy being a significant social determinant of poor health outcomes for mother and baby. It carries a stigma which is associated with maltreatment of pregnant teenagers and low uptake of maternal and child health services in this group. Despite health workers and midwives in this study insisting that low maternal age in itself should not be a cause of maternal mortality and morbidity, there are upstream factors at work which strongly influence the likelihood of very young pregnant women surviving pregnancy and thriving as mothers.

#### Abuse and abandonment

A strong narrative amongst participants was that girls who find themselves pregnant are very likely to be rejected by their families. Most of the young mothers in the study had been afraid to tell their parents, particularly their fathers, fearing physical abuse:

*So my father came home … and said if he meets me in the home he is going to shoot me with a gun since he was a policeman. (MFG1)*

*My elder brother was so annoyed that I was beaten and I was wounded on my back and the sore was there for a long time on my back. (MFG2)*

All girls reported being told to show the man who ‘owned the pregnancy’, and for those for whom the boyfriend ‘denied the pregnancy’, some returned home after a cooling off period, often mediated by their mother or another female family member. Where this was not possible, some remained away from home with friends or lived in abandoned buildings, often with no reliable source of support. When the baby’s father was prepared to ‘own the pregnancy’, an arrangement was often made for financial support to the girl’s family, or for the girl to live at the man’s house. Girls in this situation had very mixed experiences – some were treated well, and others were made to sleep on the floor and given heavy domestic duties and very little to eat.

*So I went to auntie Ami’s parlour, and I slept there on the hard tiles until nine months and was ready to have the baby. They didn’t feed me. Auntie Ami fed me once a day and let me sleep in her parlour because I did her washing and her dishes. (MFG2)*

*They threw her out of the home and she went and stayed with the boy who was an apprentice with a taxi cab driver, so they were sleeping in cars. She was cold, anaemic, not enough blood and was not eating well. She was sharing a plate of rice with her partner. She died. (WL2)*

Other studies have found that this arrangement confers higher risk of emotional and sexual abuse for the girl, and higher risks of physical and mental health problems [8, 15].

#### Increased birth complications

##### ‘Just because she’s young, it doesn’t mean she has to die’

Midwives were clear in their discussions that it is poverty and abandonment which set a girl up for maternal death rather than age per se. A strong theme which all the midwives and other health workers came back to repeatedly was ‘just because she’s young, it doesn’t mean she has to die’. They acknowledged that young girls can be less developed and need specialist midwifery and obstetric care, but were adamant that with the right care, they should be no more destined to death than an older woman.

*So it doesn’t mean she’s a teenager, she should die. (No, exactly!) If we know the risk, if she goes through the normal antenatal care, where they screen her, test the blood haemoglobin level, do head to toe assessment, palpation and all, she should receive care just as any woman who is pregnant, if we have proper, functioning systems in place. (HMFG)*

They consistently attributed adolescent maternal death to lack of care; both family care and delayed midwifery care, and were clear that one did not have to look very far upstream to discover the source of medical risks such as anaemia, malaria, pre-eclampsia and infections. Poor diet led to anaemia, and delayed care seeking meant that girls were missing out on life-saving antenatal care; blood tests for infection and haemoglobin level, antimalarial medication, iron supplements, blood pressure checks, and the health talks given at every antenatal clinic appointment:

*And because of the inexperience, they don’t know when there is a raised pressure only because they refuse to come to the antenatal … and by the time they come to the hospital they come convulsing, fitting. You see them dying. (SMI1)*

They reported teenagers often not registering at all with a health care provider in pregnancy, then presenting late in labour, often only accompanied by friends and lacking any adult support, and they related concealed pregnancies directly to maternal death.

Particularly for girls in rural areas, early marriage was seen by the midwives as a risk factor. For these girls, though living without stigma and in the safety of either their parents’ or husband’s family home, delay in care seeking was a major issue, often a result of the lack of decision-making ability of women within traditional families:

*In the provinces, when the chief wants that young grownup girl, he convinces the relatives. After marrying her she becomes pregnant. Maybe she does not even attend antenatal clinic. When it comes to delivery, complication arise. To let them refer that case to the big hospital, it’s a problem. Maybe the husband is not around, then the relatives do not have money and they do not have a say over that woman so they delay to make decision. So after they have made the final decision to take the woman, transportation! Maybe the road is not good, there is no ambulance for the patient to come to the hospital. So after the patient has arrived now in the big hospital … maybe we need blood for this patient, there is no doctor to see this patient … if she comes in with bleeding, obviously she will lose her life. (HMFG)*

Clearly, teenagers who die around the time of childbirth suffer from the same obstetric conditions which befall older pregnant women, but youth in itself should not confer additional risk when considered as part of a risk assessment – a concept which the midwives clearly articulated. Obstetric risks were perceived to be magnified by delayed care seeking and the poor physical condition in which girls go into labour.

#### Risk of death from Anaemia and PPH

In Sierra Leone, as in many other countries in Sub-Saharan Africa, most donated blood is given by family members during an emergency. Although there were some contradictory versions of how the blood bank operates in Freetown, it was commonly reported that for a patient to be given a blood transfusion, two donors were required to donate into the blood bank. Discussions with midwives from both the peripheral clinics and the referral hospital described a clear policy for minimising the risks of anaemia and post-partum haemorrhage; all women are given iron supplements and advised on diet, and haemoglobin level is checked at 36 weeks of pregnancy, when women are urged to involve family members in identifying potential blood donors:

*When the mother attends ANC, and they tell her she will need blood … she will ask her family, saying ‘look here, I’ve been going to ANC and they say I will need blood, so what I want you to do, as a family is to be thinking who will give blood’... it’s all part of birth preparedness. (HMFG)*

But with limited success:

*…well most of the time they tell them but they don’t comply, they don’t accept that there’s a problem, and then you see them running helter-skelter. (HMFG)*

Where a donor cannot be found, most participants stated that non-related donors can be paid at a high premium to donate. Where people cannot afford to pay a donor, participants reported that emergency blood is given if available but that this is not always the case, to the frustration of clinicians and community leaders:

*We don’t have much blood in the blood bank. They end up dying. We can help but we cannot give more than what we have. (SMI3)*

*Someone would have to pay for blood. I have been there on about seven occasions when young girls died there. They have that problem there. There is no free blood. People do not volunteer to give blood. Unless your relative volunteers to give blood on your behalf, if not so you would have to buy. (WL3)*

The severity of the situation was all too apparent when the first author donated her blood at the country’s main referral hospital; it was the only unit of A+ blood in the blood bank.

### Potential solutions

#### Mentoring

The literature from Sierra Leone consistently points to good parental communication, especially with mothers, as a protective factor for risky sexual behaviour and early pregnancy, but that parents lack confidence to discuss these issues [15, 16]. This study aligns with that; the country director of an international NGO addressing teenage pregnancy prevention in a rural area described ‘*overwhelming numbers of parents wanting to join in’* with their interventions to equip parents with knowledge and skills to support their children.

This was reinforced by several of the professional participants referring to their own feelings of inadequacy in this area:

*We the parents also, to some extent, we are to be blamed ... because we don’t make our children our friends. We have that communication barrier... some of us think that there is certain information that has to be hidden from these children, then they go astray as a result of that. You have to talk to your child! (HMFG)*

Many girls reported knowing very little about sex and pregnancy before they conceived. Several girls interviewed did not know they were pregnant until a family member or relative noticed their body shape changing and took them for a test. Regarding outcomes for girls once pregnant, the ability to communicate with, and good care by family members were recognised as being protective, especially in avoiding the dangers of concealed pregnancy and abortion:

*What is most dangerous thing is this abortion, because some of them are so afraid of their parents and usually it’s the boyfriend who took them to non-qualified people. (MW1)*

Regarding criteria for a young woman to move on positively with her life after a pregnancy, it is unsurprising that having supportive family relationships has been shown to be highly advantageous. Several professional participants referred to themselves or colleagues who had had a teenage pregnancy, but had progressed to a successful professional career due to good family support. Currently, all pregnant girls are forced to drop out of school as there is a controversial ban on ‘visibly pregnant’ girls attending school or sitting exams, which for some girls adds to the stigma and isolation they experience [17]. The capacity to either return to education or pursue vocational training appears to be important for mental wellbeing, avoiding rapid subsequent pregnancies, and being able to provide for their children, thus breaking the cycle of poverty. These two young mothers, enrolled at Conforti, a vocational training provider, express this:

*Now that I have given birth and decided to come for that training, I have joined the PPA so I will not give birth again. (MFG1)*

*But I bless this institution, training us. I wasn’t feeling good about myself, I just saw myself as a drop-out, I really felt it! (MFG1)*

However, the additional expense of school expenses on top of providing for an additional child is often prohibitive for families. At the time of the study, LNP was providing free training in trade-based skills to local youth, and this was considered a huge community asset:

*That (the Institute’s graduation) was very good. Some might want to learn but are not able to pay for the education. The parents might not be rich enough to pay. But if they learn a trade they will be able to provide for themselves and their children. (SCLI))*

For girls who have been abandoned by their family, or are living with the baby’s father’s family with little support, there is a recognised need for other adults in the community to act in the capacity of trusted adult. One participant, an older mother of five, had recognised this in her local community and been informally supporting pregnant girls for the previous five years. She described her strategy; ensuring the girl accesses health care whilst attempting to advocate for her within the family context:

*I had this urge, feeling and burden for children who are thirteen, fourteen, fifteen, in schools. This was bubbling inside me so … I started working with the girls that have been expelled from their family homes. I usually accompany them to talk to their parent, saying things like ‘it’s like a loaded gun - once the trigger is pulled it cannot be recalled. The only thing we can do is to take care of the girl’. I am thankful to God I have never had a victim or death... I will take the pregnant girl for check-up with a nurse making sure that everything about the pregnancy is alright then I will approach the parents. I do pray before I go to speak to the parents as some of them can be very bitter saying I would not accept the girl as she has chosen to be sexually active. Let her go and get married. I will say to them ‘this child is yours, a marriage can end but the girl will be the one who look after you when you are old.’ I will gather other people to help with the negotiation. Most times this is successful. (WL3)*

She also described her strategy to ensure that the girls had enough money to feed themselves well and buy things for their baby; supporting them to start a small business and supervising their savings.

Considering the recent EVD epidemic in Sierra Leone which left many stigmatised orphans, and the effect of the internal migration of teenagers away from families in the provinces to Freetown, this simple community-based mentoring intervention has the potential to benefit other orphaned, abandoned or otherwise isolated adolescent mothers in Freetown.

#### Community-based blood donation

Blood donation in Sierra Leone is complex, with social norms around who can donate blood to whom. It is rare for a husband to donate blood for his wife, with the woman’s birth family usually donating in this case:

*Yes, because for example if I was pregnant, my sister would be more willing to give blood for me, or my brother, so that nothing will happen to me. Rather than my husband. (NGOI1)*

*It is the dads that give blood more than the husbands. (SMI1)*

Whereas if a child needs blood, this will invariably be donated by the child’s father:

*For children, most times the father does it. The fathers are willing. But they will not give for their wife. I don’t know what the issue is. (NGOI1)*

This study identified altruism as a strong motivator for blood donation in Freetown:

*Just because when people meet me and they explain to me ‘you need to help us in this situation’, I just need to do it, because that is my only way to help them. (YMFG)*

But found that a significant barrier was HIV testing; either fear of discovering their status, or fear of their status being told to a family member:

*Yes, I think people will be afraid to be tested, in case at the end of the day the doctor says, ‘you have HIV’ (YMFG)*

*People are afraid. People don’t want to be tested because of hepatitis and HIV. So, they dodge. Some husbands they prefer to give money to donors to getting them tested, or maybe some of them know their status, they hide it from the wife, and they don’t want to expose it. (NGO1)*

This fear of stigma was all too fresh to participants after the recent EVD epidemic where affected people were ostracised by the community:

*In this country, when I say this person has HIV, they will just run away from you, it will just turn like Ebola. (YMFG)*

A further barrier was fear of the process; in a focus group of young men, there was clear lack of knowledge, with questions asked about how much blood was taken, whether it hurt, and whether your body could recover. Lastly, some common myths were raised:

*People say when you give blood you die. When you give blood, you become sick. (WLI2)*

*Some people think if they come and take one pint of blood from them they will die tomorrow. It is just fear. (YMFG)*

##### Compensation

All participants with whom blood donation was discussed believed that a donor should be compensated for what their body had lost in the process of donation:

*Even for someone to be able to give blood they should be able to have food to eat. If not so they will be anaemic. (YMFG)*

This was not considered a payment, and the donation was still classified as voluntary. For one young man who had donated blood five times to non-family members, being thanked also seemed to be an important motivator:

*The one I remember was (name). I was thinking that at the end of the day, I paid my transport to go there … I was thinking that when he got well, he would say ‘hey man thank you’, but … he couldn’t even look at me, but I had already done it, so I just let him forget about it. All the (other) four people, I’ve been happy to keep them alive, but for him, I was so angry about it, because he’s my brother (non-relative), so I was thinking he would say something like ‘thank you’. (YMFG)*

In terms of strategies to recruit non-related volunteer donors, more overt compensation is more important. For example, health staff are offered days off in exchange for a unit of blood. It was evident that various models had been tried to recruit donors, but with limited sustainability:

*There was an NGO who was paying people to donate blood. The donors were fed well. But the problem with our country we have so many groups which have been set up by so many NGOs who would come and start something, but it is for short time it does not last. (HWFG)*

A potential solution was discussed with midwives and a group of young men from LNP, of having a local blood donation register, whereby a direct communication between the clinic midwife and the co-ordinator of the register would ensure that a donor could be sent to the referral hospital directly if a woman had to be referred for bleeding. This midwife felt that not having any mechanism for compensation would be a barrier as, again, being fed a meal was a minimum expectation of donors:

*They would have to eat to replace that. We would not be able to keep this service going. It is not easy to get people to donate blood. Some people will do it once but they would make excuses the second time. They will say that they have not eaten since the morning. (MWI)*

##### Blood donation experience

The literature also shows a clear association between the first blood donation experience and the likelihood that the donor will donate again [18], and it was clear from the young man who was a multiple donor that his experience had been positive, that he had felt well cared for and given food:

*If you donate your blood, they will take care of you, explain to you how it’s supposed to be, during the blood donation, or after the blood donation. They will explain to you ‘sit down first and take a little bit of bread’. (YMFG)*

Other potential barriers to a local community register was the fear that donors might be taken for granted when there were other family members able to donate; this would need to be part of a further consultation on compensation mechanisms and eligibility.

## Discussion

### Vulnerability to teenage pregnancy

#### Transactional sex

It is evident that in Freetown, adolescent transactional sex, whether for water, money, or grades, is a social norm which is in some cases encouraged by family members, ignored by school authorities and for which legislation appears not to be effective. In many cases, expectations are placed on girls to provide for their own financial needs, and for many girls, the only option is to meet those needs with their one available resource, their bodies.

This study indicates that girls who live with extended family rather than with a parent are more vulnerable to the pressures of transactional sex and exploitation, and the literature echoes this finding [9, 15]. One 2010 study showed large anomalies between the intended reason for children being moved from rural areas to urban households and the endpoint reality for the child [15]. Sixty-seven percent of these children said they initially moved to Freetown to go to school, but only 38 % were attending school, and 60 % said they ‘worked’, the majority ‘working in the house’ [15]. Regarding pregnancy, 58 % of teenage mothers or pregnant teenagers were not living with either biological parent [15].

The more specific issue of ‘sex for grades’, where girls have sex with male teachers in exchange for passing exams or being promoted to the next year in school is also widely mentioned in the literature [8–11]. In Coinco’s 2008 qualitative study [9] focussing on the plight of ‘out-of-school’ primary-aged children, the issue is discussed in some detail, with a case-study on ‘teachers’ sexual advances’. A 2010 joint NGO report showed that some 30% of coerced or forced sex of adolescents is by teachers [11], and a 2013 study [8] acknowledges the issue of teacher coercion, but found that participants did not talk about it, concluding that increased awareness of child rights has increased the taboo around the issue with fears of recrimination and shame acting as deterrents to fuller discussion or disclosure.

Though the issue is recognised within the literature, when policy solutions are discussed, there is little acknowledgement that the pressure for sex from teachers may hinder girls’ engagement or re-engagement in education. Indeed, several narratives present the issue of sex and education as a relatively simple choice for girls; between remaining in the relationships where the transactional sex is taking place, or if support is available, returning to the school environment, with the underlying message being that school is a safe and secure environment:

‘*More targeted work is also required, for example, with regard to girls involved in sexually exploitative relationships, who need practical and personal support to exit these relationships and re-engage at school’.* (page 7) [19]

And there are even suggestions that that girls choose to pursue sex with teachers to raise their grade unfairly, rather than as an unavoidable route to completing their schooling:

*Findings reveal that girls are also engaging in transactional sex to access a range of other amenities and merits including, for example, school grades – in the aptly termed “sexually transmitted grades”* [20]*(page 18).*

This approach seems rather simplistic in the light of key informant data in this study, which indicate that, contrary to perceptions that keeping girls in school will solve the problem of teenage pregnancy, for some girls, school is a risky environment where transactional sex is a necessary evil to ensure academic success.

Much of the messaging on early sex and teenage pregnancy appear to be presenting the issue within a ‘blame frame’ [21]. Girls’ behaviour is perceived as the problem, with little being said about the role of men and boys, the impotence of the justice system, or the significant contributors within the structural environment such as the deficient water supply or lack of female teachers. Posters generally target girls themselves, urging them to avoid under-age sex and teenage pregnancy, with the implicit message that they are free agents, making choices around their sexuality and fertility. Whilst the 2014 communications strategy rightly proposes messages to both young men and women, to parents, carers and community leaders, some of the key messages belie the baseline premise that girls have agency and are in a position to choose their sexual behaviour: ‘*Abstinence is the safest way to avoid pregnancy and diseases. Young women who choose this path should be praised and encouraged’.* [16]*(Page 41).*

Some studies rightly identify that girls have transactional sex in order to access clothes and belongings which would be inaccessible without it [20]. There is a danger that this too can feed into the blame narrative, as though having sex for basic needs is more acceptable than having sex for ‘luxuries’. Again, this oversimplifies the lived experiences of girls in Freetown. Seminal literature on ‘the body as an asset’ asserts that for a poor farmer or factory worker, the physical body is the one asset a person has to produce wealth [22]. For a girl in Freetown, her body is such an asset, and equipping herself with the right clothes, hair and accessories could be seen as a way of maximising on her potential to pay for ‘necessities’ such as schooling, which is widely viewed as the only exit from poverty. For example, a mobile phone could be viewed as a luxury, but with the ‘body as an asset’ lens, a mobile phone is to a teenage girl as a scythe is to a farmer – an essential tool to maximise one’s potential to secure a livelihood.

Data suggests that sexual exploitation by teachers is not restricted to senior secondary schools, but also directed towards primary age girls in early puberty with no transaction apparent. However, the language used to discuss this issue lacks clarity, with phrases such as ‘institutionalised child abuse’, or even ‘child abuse’ not being part of the dialogue. One key participant, describing his discomfort with the situation where a teacher keeps a pubescent girl after class to ‘touch her breasts and buttocks’ proposed tentatively that ‘this might be called abuse?’ The exception to this was a child protection worker pushing for a teachers’ charter and awareness-raising amongst students on the law around sexual abuse and child rights. In the recent years of the implementation of the national strategy, efforts have been made to educate communities about gender-based violence and child rights, but there is clearly more work to be done in this area.

In many ways, this lack of conviction about what constitutes child abuse is unsurprising, since the waters are muddied by the strong gender norms which normalise early marriage and motherhood, often viewed by families as a way to prevent the risk of the stigma of teenage pregnancy outside of marriage, and boys’ education is often seen as a more worthwhile investment than girls’, with the expectation that early motherhood will limit educational potential. Though reported elsewhere [15], there was no evidence in this study of boys being sexually exploited by teachers. However, they are not exempt from ‘money for grades’ by both male and female teachers, and often do hard manual labour outside of school to pay for additional charges. All these factors undermine the education system and therefore economic growth and development. A unique finding in this study was the use of boys by teachers to scout out and groom girls, promoting and reinforcing a gendered stereotype of adult male sexual behaviour to boys through negative role modelling.

Training and employing more female teachers is recognised as important to turn the tide in this issue, but it seems that this very strategy is undermined by the issue itself; only 12% women aged 20–24 in Sierra Leone are educated to secondary level or higher, compared with 20% of men [1] and the data suggests that sex for grades is also a common barrier for women in university. This under-education of girls has serious implications for the health and economic wellbeing of the country as a whole; an international study shows that when women and girls earn income, they reinvest 90% of it into their families, compared with only 30 to 40% for a man [23].

Regarding the ‘water for water’ issue, the same message persists; posters encouraging girls to avoid early sex and stay in school are strategically placed near water wells [16]. The irony of this message is hard to miss when one imagines a girl considering her options of staying in the water queue and missing school, or of bypassing the queue for sex to ensure she attends school or sits an exam. A more upstream solution, to upgrade the failing water supply problem, is not mentioned as a potential strategy in policy documents.

### Prevention of adolescent maternal death

#### Abandonment

The association between poverty and adolescent maternal death was pervasive in this study. This was particularly clear within the themes about the treatment of girls once pregnant, with clear differences between social groups. Poorer families normally see the girls as the responsibility of the father of the baby, and an opportunity to gain some financial support, even if he is a teacher or older married man. Support deals are often brokered by community leaders for the man to support the girl during the pregnancy and pay for the child’s education, even if she stays with her family. Girls who are forced to marry or to live at the man’s house are often treated badly, exposed to further risks of STIs, malaria, anaemia and the additional risk of second pregnancy; compounding mortality risk.

This is in stark contrast to girls from professional families. Many of the professional women within health services, NGOs and government departments described being introduced to a colleague’s son or daughter who was clearly the result of a teenage pregnancy. One participant related this phenomenon to Krios, an ethnic group within Freetown who tend towards professional occupations. She described how girls as young as eleven would be taken to a different community (‘*to the hills’*) and given caesarean sections, only to return to school without the knowledge of their peers.

This practice of the family concealing and dealing with the pregnancy and sending the girl back to school is very difficult for poorer families, yet one local woman advocated strongly for a similar mindset, articulating a view not commonly heard in poorer communities; of families continuing to treat the girl as a child, and taking the baby as a sibling of the daughter. She stressed the importance of helping girls to develop aspirations, and the need for strong role models. Much has been written about the influence of positive role models in the Sierra Leone context [8, 9, 15], and examples such as this one point to grass-roots advocacy as a powerful tool for change in tackling the inherent social inequalities of teenage pregnancy outcomes.

#### Delayed care seeking

The literature and current study consistently point to delayed care seeking as a key risk factor for adolescent maternal death [24]. Fear of insensitive or harsh care by health workers, lack of confidentiality and being stigmatised by other women are all cited as factors [25]. Sierra Leone has a mixture of government clinics, private clinics and a referral hospital. Several participants perceived that care in private clinics is more respectful, and women who can afford it seek out private care for this reason, despite care being generally safer within the state system.

*I’m not going to go and give my money and somebody will yell at me. I will pay money where I am respected. I don’t care if I don’t eat for the day, but I will pay my money, as long as I’m respected and somebody speaks nicely to me.* (NGO)

*Well in the private hospitals, people go there for the respectful care, but also they should know that though respectful is important, the skill also is very important. Because it’s the skill that will save your life.* (SM)

Without the means to pay for private care, adolescents often choose to give birth with a traditional birth attendant (TBA) for similar reasons.

*And even for the TBAs, right, the TBAs will never never, in as much as they have their own issues, they will never yell at a woman when they go to see them in their house. As soon as they see them, they are ‘how are you?’, even when they don’t have all the skills.* (NGO)

In addition, despite health care for pregnant and lactating women and children under five being free since 2010, there is a perception that government clinics still charge for their services, and in reality, informal charges are still common.

Viewing the situation from the health worker’s point of view, training for health workers is rife with corrupt practices such as ‘under the table’ payments to invigilators, for forms to be processed, and for graduation, and health workers often leave their training in huge debt; this inevitably plays out in the clinics and hospitals. A key NGO participant leading a community development project aimed at reducing maternal mortality through community engagement, highlighted the importance of a strong advocate as a protective factor against these informal charges; a respected individual who could challenge disrespectful care or corrupt practices. Girls who are only supported by peers or young boyfriends are particularly vulnerable and may not return to a clinic or hospital setting if their first experience is disrespectful or corrupt, risking complications and death. One young mother who experienced abusive care in labour vowed she would never use the services again, and joked that she would protect herself with six ‘captain bands’.

Midwives talked about how the combination of FGM, early marriage, polygamous households and delay in seeking care mean that girls from rural areas often arrive at the referral hospital in Freetown at the point of death. Their general feeling is that physically, girls ‘shouldn’t have to die just because they are young’, but lack of agency means that girls have little control over their fertility or health seeking, and this makes them much more vulnerable. Midwives expressed strong emotions when talking about the helplessness they feel when girls die needlessly. One interviewee reframed the harsh treatment of women who seek health care late as an expression of frustration and helplessness as *‘when they yell at you it’s because they love you and don’t want these things to happen to you’.*

There were numerous examples in the data of midwives going ‘above and beyond’ for women, including doing a home visit to site implants for girls who were too ashamed, donating blood for a dying woman, and paying for a taxi to transport a woman to hospital who was bleeding. This is not to say that the promotion of respectful free care is unimportant, but gives another perspective to the ‘disrespectful care’ narrative which needs to be considered in the bigger picture of resolving the issue. Starting a career without the debt from bribes paid to move through the training process would undoubtedly affect the issue of informal charges, for example.

### Potential solutions

#### Mentoring

Historically, sex and relationship education in school has been limited to lectures by teachers or nurses on contraception use, with little opportunity for real discussion with adults [8, 16, 19], and studies show that adolescents are strongly influenced in their attitudes to sex, and gain most of their information about sex from pornography and other media [8, 20].

Although evidence which specifically relates to mentoring of young mothers is sparse [26, 27], with most research looking at black American adolescents, limiting applicability to the African setting, there is strong evidence that connectedness with an adult, be it a parent or a non-parental adult, appears to be foundational for adolescent health and well-being more generally [26, 28], including delaying rapid second pregnancies [27]. Regarding studies in SSA, Coinco asserts that a protective factor for adolescents against risky sexual behaviour and early pregnancy in Sierra Leone is having a consistent trustworthy adult or role model involved in their lives [15], and studies in South Africa and Uganda demonstrate that AIDS-orphaned children and adolescents in a natural mentoring relationship showed significantly lower distress and mental health factors (child abuse, social discrimination, anxiety, and depression) than those not in a mentoring relationship [29, 30]. A review of the determinants of delivery service use identified low maternal age as a determinant for not accessing skilled care for delivery [31].

Within this current study, issues of abandonment leading to poor mental health, poor health-seeking behaviour and lack of knowledge about pregnancy, birth and baby care all indicate that a mentoring scheme may be effective, and evidence from other community projects indicate that having an advocate when accessing health care can mitigate against some of the barriers such as disrespectful care and informal charges (personal communication).

Following the findings of this study, a pilot mentoring scheme for younger and more vulnerable pregnant teenagers is currently underway, in order to establish feasibility to trial a scaled-up intervention. Mentors have four key roles; helping girls to re-establish family connection and support; encouraging health seeking behaviours and advocating for respectful care at clinic, in labour and in the postnatal period; providing practical advice and support with parenting; and providing support and training in a small business and to re-enter education or vocational skills training. Some outputs will be quantitative measures such as the number of antenatal visits, uptake of tetanus toxoid, uptake of prophylactic medication, sleeping under a bed net, and uptake of infant immunisations. Others will be qualitative in nature; the impact of the mentoring relationship on the young person’s mental health, the impact of having an advocate on experience of antenatal care, and the impact of mentoring on family support mechanisms.

#### Community-based blood donation

The Guidance from the World Health Organisation (WHO) and the President’s Emergency Plan for Aids Relief (PEPFAR) recommends that countries aim by 2020 for 100% blood to be donated by non-remunerated volunteers rather than family members, primarily for reasons of sustainability and safety of the service [32]. The need for an understanding of the SSA context and how this differs from the primarily European and American contexts where most of the research which underpins the guidance is carried out, was emphasised in two recent literature reviews around blood donation behaviour in SSA [33, 34]. Both reviews identify altruism as the common primary motivator for donating blood, whether to a stranger or a family member, but show that other motivators and barriers vary between countries. For example, the opportunity to have a health check and discover and treat any infections was considered a motivator in a study from South Africa, but a deterrent in studies from many of the other SSA countries. Regarding barriers to donation, a mixture of cultural and religious beliefs, myths about the dangers of donation, and lack of information about the process were all cited, and their analyses indicate the need for programming at a national or even tribal level rather than global or regional level. For example, in several of the thirty-five studies in one review, there was a belief that giving blood could cause male impotence [33]; where this is known, interventions with older repeat male donors with children as donor champions could be strategically effective.

Regarding the distinction between regular volunteer donors and those who donate to family members in an emergency, the evidence behind the rationale of safety and sustainability of the former has been challenged, with further analysis of the data showing no difference in safety and a two to five times increased cost per unit for this policy [35].

In a culture where blood is not readily available and blood donors must be brokered within the family setting, it is unsurprising that young girls in Freetown who have been rejected by their families are at higher risk of death from bleeding than women with higher levels of social capital. This is in addition to risks due to increased anaemia from poor diet and limited antenatal care. Social norms dictate that it is close relatives rather than husbands or partners who donate to women, putting young girls in an even more vulnerable situation. If a blood donor is not available, blood must be purchased which is likely to be outside the reach of more isolated teenagers.

One potential solution being considered here is to set up a local register of donors from a discreet community, linking directly to the three PHUs in that community, where donors who have already been screened are called upon in an emergency. This system would engender a sense of ownership, and allow for compensatory mechanisms between families and donors not possible within a wider donation programme. With an asset-based lens, the community in question has particular favourable assets for such a scheme; the hub of this community is a faith-based project set up during the civil war to rescue child soldiers, and which now accommodates and trains young people, based on common values such as altruism and caring for the poor. There is strong body of literature on the benefits of maximising on faith-based groups as sites for public health interventions [36], with potential for scale-up in similar communities.

In this study, compensation to eat and travel to the blood bank were considered minimum requirements to donate, with this donation still being considered voluntary and altruistic. Since paying large sums to ‘professional’ blood donors outside the hospital was commonplace, a system where the family ‘gifts’ the donor a modest amount of money to travel and eat could be reasonably considered as a sustainable model. The importance of recognition was also seen as important, and if the system was being managed within a discreet community there would be scope for developing this particular incentive.

The biggest barrier to blood donation was the fear of a positive HIV status, with the belief that this was a death sentence and would lead to social ostracization. Other barriers were fear of the process and fear that the donor would be unwell after donation. More exploration needs to be done with this community about the best ways of addressing these barriers. For example, in the focus group with young men from LNP, the fact that one young man had donated blood five times and described the process as *‘like having a drip – you’ve all had a drip!’* seemed to quickly dispel the fear in the whole group. Equally, meeting a person living with HIV, or hearing stories from community members whose lives have been saved by a voluntary donor could be important strategies for recruitment of local donors, and would need to be further explored.

## Conclusion

This study has explored the causes of high maternal death in younger teenagers in Eastern Freetown, Sierra Leone using interviews and focus groups with a wide range of participants including adolescent mothers, midwives, teachers, policy makers, third sector groups and community leaders. Three main areas of discussion were vulnerability to teenage pregnancy, vulnerability to maternal death once pregnant, and possible interventions to address these vulnerabilities.

Under the more commonly explored issue of vulnerability to teenage pregnancy, it is evident that girls use transactional sex to access a range of resources including school fees, academic success, time, and money for food and clothing. Girls from poorer families, and girls cared for in a family other than their birth family appear to be particularly vulnerable. Narratives around teenage pregnancy assume a degree of agency which is not found in this data, and strategies discouraging early sex appear to emphasise giving of information and behaviour change at the expense of more upstream interventions such as improved water systems, more female teachers and better enforcement of laws on sexual abuse of children.

Regarding vulnerability to maternal death, concealing pregnancies and abortions, and being abandoned by families are significant risk factors. Midwives strongly concurred that adolescence in itself should not put girls at higher risk if maternity care is accessed in a timely manner, but in reality, the lack of adult care and supervision and lack of decision-making power put young teenagers at higher risk of death than older women who have more knowledge, social capital and agency. These factors were evident in the issue of blood donation, where delayed care seeking, lack of knowledge, and the absence of willing blood donors all put young girls at increased risk of death from bleeding.

Two potential interventions emerged from the study; a volunteer mentoring programme, and a locally managed blood donation register, neither of which have been documented previously. Of these, the pilot mentoring scheme for younger and more vulnerable pregnant teenagers is currently underway. Mentors will encourage early uptake of antenatal care and use of a trained birth attendant, will help to maintain or re-establish family support, will support moving on to training or return to school, and will support the young woman to start a small income generation activity. A feasibility study of this pilot project will use qualitative methods to determine whether the scheme is acceptable within the community, accessible to the most vulnerable girls, and whether the data collection tool is fit for purpose. This study will then form the basis of a larger trial to establish effectiveness in terms of mortality and wellbeing outcomes. If effective and scalable, this intervention has the potential to affect outcomes on a large scale for adolescents and their children across Sierra Leone and beyond.

## Abbreviations

CBO: Community Based Organisation
EVD: Ebola Virus Disease
GoSL: Government of Sierra Leone
HDI: Human Development Index
LiST: Lives Saved Tool
LNP: Lifeline Nehemiah Project
MMR: Maternal Mortality Ratio
NGO: Non-Governmental Organisation
PEPFAR: President’s Emergency Plan for Aids Relief
PHU: Peripheral Health Unit
SLDHS: Sierra Leone Demographic Health Survey
SSA: Sub-Saharan Africa
TBA: Traditional Birth Attendant
WHO: World Health Organisation

## Footnotes

1 The six pillars are:

- Improved policy and legal environment to protect adolescents’ and young people’ rights
- Improvement of access to quality sexual reproductive health, protection and education services for adolescents and young people
- Comprehensive age-appropriate information and education for adolescents and young people
- Communities, adolescents and young people empowered to prevent and respond to teenage pregnancy
- Coordinating, monitoring and evaluation mechanisms in place, allowing proper management of the strategy
